# Circulatory trajectories after out-of-hospital cardiac arrest: a prospective cohort study

**DOI:** 10.1186/s12871-021-01434-2

**Published:** 2021-09-08

**Authors:** Halvor Langeland, Daniel Bergum, Trond Nordseth, Magnus Løberg, Thomas Skaug, Knut Bjørnstad, Ørjan Gundersen, Nils-Kristian Skjærvold, Pål Klepstad

**Affiliations:** 1 Department of Anesthesiology and Intensive Care Medicine, St. Olav’s University Hospital, Trondheim, Norway; 2 Institute of Circulation and Medical Imaging, Faculty of Medicine and Health Sciences, Norwegian University of Science and Technology (NTNU), Trondheim, Norway; 3 St. Olavs Hospital HF, Avdeling for Thoraxanestesi Og Intensivmedisin, Postboks 3250, 7006 Trondheim, Torgarden Norway; 4 Department of Anesthesia, Molde Hospital, Molde, Norway; 5 Department of Emergency Medicine and Pre-Hospital Services, St. Olav’s University Hospital, Trondheim, Norway; 6 Clinical Effectiveness Research Group, Institute of Health and Society, University of Oslo, Oslo, Norway; 7 Department of Transplantation Medicine, Oslo University Hospital, Oslo, Norway; 8 Department of Cardiology, St. Olav’s University Hospital, Trondheim, Norway

**Keywords:** Out-of-hospital cardiac arrest, Post-cardiac arrest syndrome, Circulation, Hemodynamic, Cluster, Sequence analysis

## Abstract

**Background:**

Circulatory failure frequently occurs after out-of-hospital cardiac arrest (OHCA) and is part of post-cardiac arrest syndrome (PCAS). The aim of this study was to investigate circulatory disturbances in PCAS by assessing the circulatory trajectory during treatment in the intensive care unit (ICU).

**Methods:**

This was a prospective single-center observational cohort study of patients after OHCA. Circulation was continuously and invasively monitored from the time of admission through the following five days. Every hour, patients were classified into one of three predefined circulatory states, yielding a longitudinal sequence of states for each patient. We used sequence analysis to describe the overall circulatory development and to identify clusters of patients with similar circulatory trajectories. We used ordered logistic regression to identify predictors for cluster membership.

**Results:**

Among 71 patients admitted to the ICU after OHCA during the study period, 50 were included in the study. The overall circulatory development after OHCA was two-phased. Low cardiac output (CO) and high systemic vascular resistance (SVR) characterized the initial phase, whereas high CO and low SVR characterized the later phase. Most patients were stabilized with respect to circulatory state within 72 h after cardiac arrest. We identified four clusters of circulatory trajectories. Initial shockable cardiac rhythm was associated with a favorable circulatory trajectory, whereas low base excess at admission was associated with an unfavorable circulatory trajectory.

**Conclusion:**

Circulatory failure after OHCA exhibits time-dependent characteristics. We identified four distinct circulatory trajectories and their characteristics. These findings may guide clinical support for circulatory failure after OHCA.

**Trial registration:**

ClinicalTrials.gov: NCT02648061

**Supplementary Information:**

The online version contains supplementary material available at 10.1186/s12871-021-01434-2.

## Introduction

Circulatory failure frequently occurs after out-of-hospital cardiac arrest (OHCA) and is part of the post-cardiac arrest syndrome (PCAS). It is believed to be secondary to myocardial dysfunction and systemic inflammation due to global ischemia–reperfusion injury [1].

Three studies provided detailed descriptions of circulatory patterns in subgroups of OHCA patients by measuring cardiac output (CO) and systemic vascular resistance (SVR) at specific time points [2–4]. The circulatory instability was characterized by a low cardiac index and filling pressures, and the median time to onset was approximately seven hours [2]. After 24 h, the cardiac index increased, but superimposed vasodilatation delayed the discontinuation of vasopressors and fluid treatment [2].

The American Heart Association (AHA) guidelines for resuscitation and post cardiac arrest treatment recommend tailoring treatment to the specific subgroups of patients who most likely benefit from the interventions [5]. Sequence analysis is a method to describe and analyze patient development over time and to identify clusters of patients with similar trajectories [6, 7]. Three cohort studies of patients with sepsis have utilized this method to identify patients with similar “clinical phenotypes” [8–10]. To date, no studies have used sequence analysis in patients after OHCA.

The International Liaison Committee on Resuscitation (ILCOR) indicates several knowledge gaps concerning the optimal treatment of PCAS. One of these knowledge gaps is how to best deliver circulatory support after cardiac arrest [11].

The aim of this study was to analyze circulatory development after OHCA. To better understand the different “circulatory phenotypes” in PCAS, we identified clusters of patients with similar trajectories and potential predictors for cluster membership.

## Methods

### Study design

This was a prospective single-center observational cohort study including patients with OHCA who were admitted to the hospital with return of spontaneous circulation (ROSC). Patients were included between January 2016 and November 2017.

### Setting

St. Olav’s University Hospital is a 938-bed tertiary hospital in Trondheim, Norway, serving a population of approximately 700,000 [12].

### Eligibility

Both comatose and awake adults admitted to the ICU with ROSC after OHCA were assessed for eligibility. Exclusion criteria were age < 18 years, pregnancy, assumed septic or anaphylactic etiology of cardiac arrest, transfer from another hospital, decision to limit life-sustaining therapy upon arrival, acute cardiothoracic surgery, intervention with extracorporeal membranous oxygenation (ECMO) or a ventricular assist device (VAD) before arrival in the ICU.

### Study period

Patients followed the study protocol from the time of admission and the subsequent five days, or until the patient died, underwent ECMO/VAD/acute cardiothoracic surgery, life-prolonging therapies were limited, or were transferred to a general ward or another hospital. Day zero had variable length depending on the time of inclusion, whereas day one started the following morning at 06:00.

### Study procedure

All comatose patients without contraindications received a pulmonary artery catheter (PAC) (Swan-Ganz CCombo, Edwards Lifesciences, USA) for continuous central hemodynamic measurements. Twice daily, we calibrated the PAC oxygen saturation sensors and measured wedge pressure.

The electronic critical care management system (Picis CareSuite, Optum Inc., USA) recorded heart rate, blood pressure, peripheral transcutaneous oxygen saturation, fluid balance, medications and respiratory support. In patients with PAC, the system collected cardiac output, pulmonary artery pressure, mixed venous saturation, and calculated systemic vascular resistance. From the prehospital report and hospital record, we registered data according to the Utstein template [13], Charlson Comorbidity Index [14], and information on assessment and treatment.

We calculated the Simplified Acute Physiology Score 2 (SAPS-2) 24 h after admission and Sequential Organ Failure Assessment (SOFA) scores daily [15, 16]. After 30 and 180 days, we obtained survival status and cerebral performance category (CPC) from the hospital records [17].

Thrombocyte count and creatinine and bilirubin serum concentrations were measured at inclusion and every day at 06:00 during the study period. Every six hours, we obtained an arterial blood gas sample.

### Post-cardiac arrest care and cardiovascular support

Comatose patients were cooled (36 °C) for 24 h. Patients with a suspected ischemic etiology of cardiac arrest received coronary angiography and percutaneous revascularization.

In the presence of hypotension and clinical signs of tissue hypoperfusion, circulation was optimized through fluid and vasopressor administration based on the department’s guidelines on circulatory support. A detailed description of the post-cardiac arrest care in this study has been published [18].

### Circulatory state classification

Patients’ circulatory measurements were classified every hour into one of three circulatory states: ‘undisturbed’, ‘disturbed’ or ‘severely disturbed’, based upon the least favorable measurement. We used predefined values of mean blood pressure, heart rate, lactate concentrations, fluid resuscitation, vasoactive medications and the need for mechanical circulatory support (Table 1) [18]. There is no consensus on the definition or classification of circulatory instability. For this reason, hemodynamic variables and corresponding cutoff values were based upon general guidelines, clinical relevance and availability during routine monitoring of critically ill patients. Central venous oxygen saturation was initially included in the classification but was omitted because therapeutic infusions hindered sufficiently frequent measurements [18].

**Table 1:** Circulatory states^a^

Variables	Undisturbed	Disturbed	Severely disturbed
Mean arterial pressure, mmHg	≥ 65	45–64	< 45
Heart rate, beats per minute	51–100	< 50, 101–130	≤ 40, > 130
Lactate, mmol/l	< 2	2–4	> 4
Fluid resuscitation, l/hours	< 0.5	0.5–1.9	≥ 2
Norepinephrine, μg·kg^−1^·min^−1^	< 0.1	0.1–0.29	≥ 0.3
Dobutamine, μg·kg^−1^·min^−1^	No	< 10	≥ 10
Vasopressin	No	No	Yes
Epinephrine	No	No	Yes
Levosimedan	No	No	Yes
Aorta balloon pump	No	No	Yes

^a^Every hour a patient was classified as having undisturbed, disturbed or severely disturbed circulation according to the least favorable measurement at that time (e.g., isolated mean arterial pressure of 40 mmHg is sufficient to classify a patient as having severely disturbed circulation)

### Statistical analysis

We assessed patients’ transitions among the circulatory states of ‘undisturbed’, ‘disturbed’ or ‘severely disturbed’ using a multistate model [19]. For instance, a patient in a ‘disturbed’ state may transition to either an ‘undisturbed’ or ‘severely disturbed’ state [20]. Furthermore, the state transitions also describe a sequence of states, i.e., a “trajectory”, for each patient. In addition, if the patient did not complete the study period, we considered the reason for incompletion to be informative and coded it into one of three “absorbing states”, i.e., ‘death’, ‘still treated in ICU’ or ‘transferred to ward in stable circulatory condition’.

We used sequence and cluster analysis to analyze patient trajectories. In this process, an algorithm uses pairwise optimal matching and Ward’s minimal variance method to group the sequences hierarchically into clusters of similar trajectories [6]. In optimal matching, similarity between trajectories is measured by the penalty cost for editing a sequence into another, and the result of all pairwise matches is recorded in a matrix. As recommended, the penalty cost of insertion or deletion was set to 1, and the cost of substitution was based on the transition rate [6]. Ward’s method evaluates all possible cluster combinations to build a hierarchy of clusters with the least variance bottom-up until the preset number of clusters is identified [6]. Based on previous studies on intensive care populations, we aimed to identify four clusters [9, 21].

We used ordered logistic regression to estimate the odds ratio for cluster membership based on independent factors related to patient demographics, resuscitation episode and status at hospital admission. In ordered logistic regression, the odds ratios among clusters are equal, and the odds ratio is interpreted as the odds of a higher (here: worse) cluster membership. Based on predictors from previous studies, we included age, comorbidity, shockable initial rhythm, time to ROSC, base deficit at admission and circulatory shock at admission to predict cluster membership and the anticipated circulatory trajectory [22, 23].

Data were extracted and analyzed using MATLAB software (Mathworks Inc., USA). Statistical analyses were performed using Stata version 16.0 (StataCorp LCC, USA) and R version 3.6.0 [24]. The R package “TraMineR” was used for sequence analysis and visualization of both individual sequences of circulatory states and transversal distributions of circulatory states during the ICU period [6].

### Sample size

This is a descriptive study, and no formal sample size calculation was performed [25].

### Ethics approval and consent to participate

The Regional Committee for Medical and Health Research Ethics, Central Norway Health Region (REK Midt, No. 2015/1807) approved this study. Written informed consent was obtained from either the patient or next-of-kin if the patient was unable to consent.

## Results

### Demographics

Among 71 patients admitted with ROSC after OHCA during the study period, 65 were assessed for eligibility, and 50, 42 of which were comatose, were included in the study (Supplementary Figure 1). Fifteen patients were excluded for the following reasons: seven because life-sustaining treatment was withdrawn upon arrival at the hospital, two had septic causes of cardiac arrest, two were not in need of intensive care treatment, two patients received VAD, one received ECMO and one patient underwent immediate cardiothoracic surgery. PAC was inserted in 30 of the included comatose patients. The primary contraindications were bleeding diastasis after percutaneous coronary intervention, implantable cardioverter-defibrillator or technical difficulties.

Baseline demographic data are presented in Table 2. Mean patient age was 62.7 (standard deviation (SD) 15.3) years, 40 (80%) were males, and the median Charlson Comorbidity Index score was 3 points (first to third quartiles (Q1–Q3): 2–4). In 42 (84%) patients, cardiac arrest was of cardiac etiology, and ventricular fibrillation was the initial rhythm in 37 (74%) patients. Forty-four (88%) patients received bystander cardiopulmonary resuscitation. The median ambulance response time was 9.5 (Q1–Q3: 5–13.5) minutes. ROSC was achieved after a median of 24 (Q1–Q3: 14–32) minutes from the time of the emergency call.

**Table 2:** Demographics and outcomes

Patient characteristics and outcomes	All	Comatose	Awake
	*N* = 50	*n* = 42 (84%)	*n* = 8 (16%)
**Demographics**			
Age, years, mean (sd)	62.7 (15.3)	64.8 (14.5)	51.8 (14.5)
Male sex, no. (%)	40 (80)	35 (83)	5 (62)
Body mass index, mean (sd)	27.5 (6.6)	27.7 (7)	26.4 (2.3)
**Medical history**			
Charlson comorbidity index, median (Q1–Q3)	3 (2–4)	3 (2–5)	2 (1–3)
Cerebral performance category, median (Q1–Q3)	1 (1–1)	1 (1–1)	1 (1–1)
**Cardiac arrest**			
Location, no. (%)			
Place of residence	19 (38)	16 (38)	3 (37)
Public place	21 (42)	16 (38)	5 (63)
Other	10 (20)	10 (24)	0 (0)
Bystander witnessed, no. (%)	42 (84)	34 (80)	8 (100)
Bystander performed CPR, no. (%)	44 (88)	37 (88)	7 (87)
First monitored rhythm, no. (%)			
Shockable			
Ventricular fibrillation	37 (74)	30 (71)	7 (88)
Ventricular tachycardia	2 (4)	1 (2)	1 (12)
Nonshockable			
Asystole	4 (8)	4 (10)	0 (0)
Pulseless electric activity	7 (14)	7 (17)	0 (0)
Number of defibrillations, median (Q1–Q3)	2 (1–4)	2 (1–4)	1 (1–2)
Time from cardiac arrest to event, median (Q1–Q3)			
Start of basic life support—min	1 (1–2)	1 (1–2)	1 (1–2)
Start of advanced life support—min	9 (5–13)	10 (5–15)	5 (2–7)
Return of spontaneous circulation—min	24 (14–32)	26 (19–35)	8 (4–14)
Adrenaline—mg, median (Q1–Q3)	0 (0–2)	1 (0–3)	0 (0–0)
Presumed etiology, no. (%)			
Cardiac	42 (84)	34 (81)	8 (100)
Asphyxia	5 (10)	5 (12)	0 (0)
Other	3 (6)	3 (7)	0 (0)
Certain pulmonary aspiration, no. (%)	9 (18)	9 (21)	0 (0)
**At admission**			
Body temperature, °C, mean (sd)	35.3 (1.1)	35.2 (1.0)	36.5 (0.6)
In circulatory shock^a^, no. (%)	18 (36)	18 (42)	0 (0)
Arterial blood gas, mean (sd)			
pH	7.18 (0.14)	7.17 (0.15)	7.28 (0.05)
pCO_2_, kPa	6.2 (1.8)	6.4 (1.9)	5.2 (0.7)
Base excess, mmol/l	-9.4 (7.4)	-9.7 (7.7)	-7.6 (5.2)
HCO_3_, mmol/l	17.9 (4.5)	17.7 (4.4)	19.1 (4.7)
Lactate, mmol/l	6.7 (4.2)	6.9 (4.5)	5.9 (2.7)
Oxygen saturation, %	92.1 (9.4)	91.5 (10.1)	95.2 (3.1)
Acute intervention, no. (%)			
Angiography	26 (52)	21 (50)	5 (63)
Percutaneous coronary intervention	21 (42)	17 (40)	4 (50)
Computer tomography scan	18 (36)	16 (38)	2 (25)
Pulmonary artery catheter	30 (60)	30 (71)	0 (0)
Therapeutic hypothermia protocol initiated	28 (56)	28 (67)	0 (0)
Prone position, no. (%)	2 (4)	2 (5)	0 (0)
Simplified Acute Physiology Score II ^b^, mean (sd)	62 (19)	68 (12)	28 (9)
**Length of stay**			
ICU, days, median (Q1–Q3)	6 (3–12)	8 (4–12)	2 (2–3)
Ventilator time, hours, median (Q1–Q3)	64 (12–162)	93 (28–173)	0 (0–0)
Hospital, days, median (Q1–Q3)	14 (7–20)	14,5 (7–20)	13 (6–16)
**Outcome, 180 days**			
Mortality, n (%)	16 (32)	16 (38)	0 (0)
Cerebral	10 (20)	10 (23)	-
Circulatory	2 (4)	2 (5)	-
Respiratory	0 (0)	0 (0)	-
Multiorgan failure ^c^	3 (6)	3 (7)	-
Other/unknown	1 (2)	1 (2)	-
Cerebral performance category, n (%)			
I—Normal	22 (44)	22 (52)	8 (100)
II—Moderate disability	2 (4)	2 (5)	0 (0)
III—Severe disability	2 (4)	2 (5)	0 (0)
IV—Coma or vegetative state	0 (0)	0 (0)	0 (0)
V—Brain death	16 (32)	16 (38)	0 (0)

Comatose indicates patients who were intubated and gave no contact (GCS < 8). Awake patients were responsive and followed instructions

*ICU* Intensive care unit, *GCS* Glasgow coma scale, *SD* Standard deviation, *Q1–Q3* first to third quartiles

^a^Systolic blood pressure < 90 mmHg or in need of fluids and/or vasopressors to maintain systolic blood pressure > 90 mmHg

^b^After 24 h

^c^If failure of two or more organ systems led to death

### Clinical circulatory variables

**Fig. 1:**
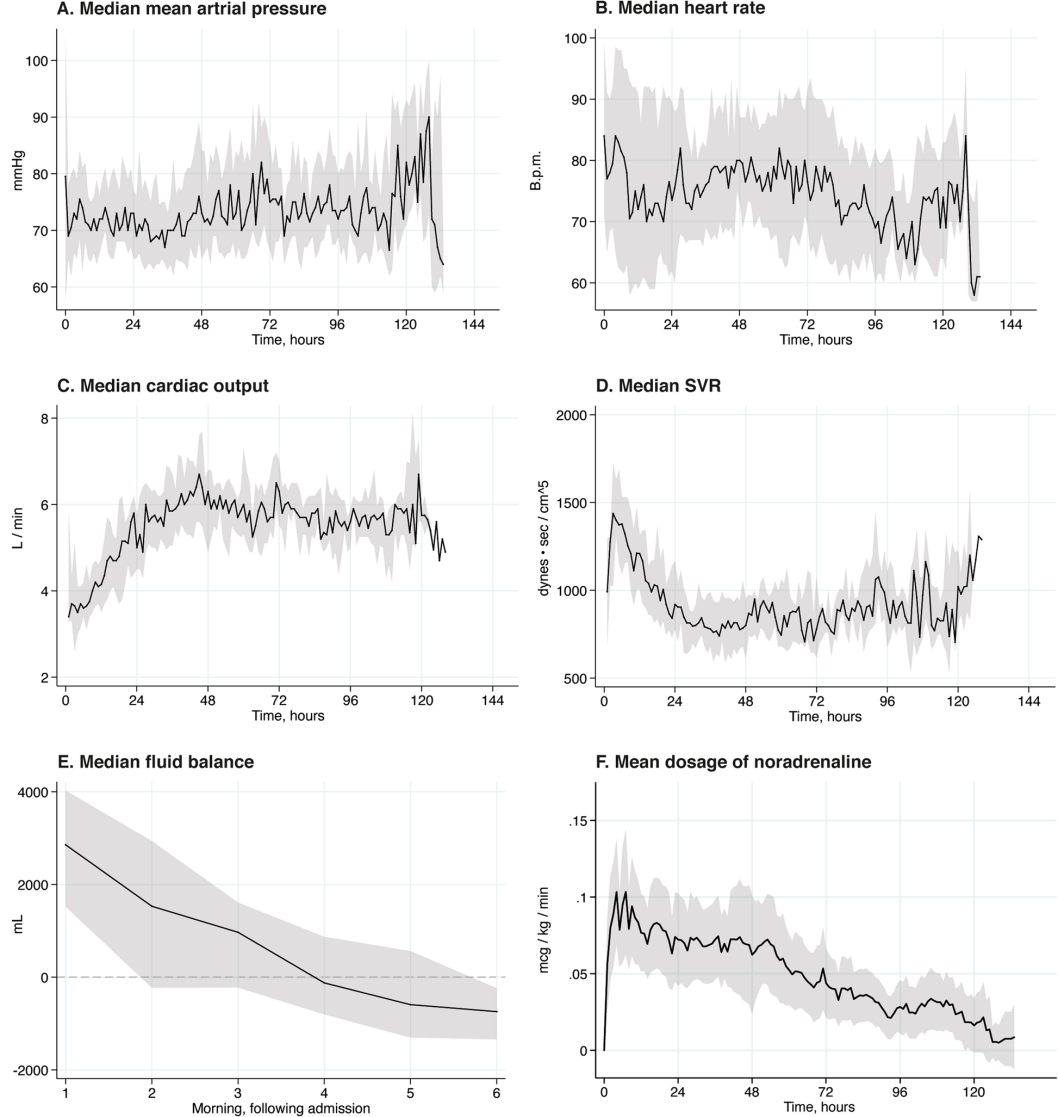
Clinical circulatory variables. **A** Median value of the mean arterial pressure with interquartile range indicated by the shaded area. **B** Median heart rate with interquartile range indicated by the shaded area. **C** Median cardiac output with interquartile range indicated by the shaded area. **D** Median systemic vascular resistance with interquartile range indicated by the shaded area. **E** Median fluid balance with interquartile range indicated by the shaded area. Fluid balance was calculated every morning. **F** Mean dose of noradrenaline with 95% confidence interval indicated by the shaded area. B.p.m: beats per minute. SVR: Systemic vascular resistance

The median mean arterial pressure (MAP) was stable at approximately 70 mmHg and increased slightly after 24 h, whereas the median heart rate varied between 70 and 80 beats per minute (Fig. 1A and B). Mean pulmonary artery wedge pressure was stable between 11 and 13 mmHg, whereas median mean pulmonary arterial pressure (MPAP) was stable between 23 and 25 mmHg. The initial median CO was 2.8 L·min^−1^, with a median SVR of 1400 dynes·sec^−1^·cm^−5^ (Fig. 1C and D). Thereafter, the median CO increased, and the median SVR decreased until 48 h, when the median CO stabilized at approximately 6 L·min^−1^ and the SVR stabilized at approximately 800 dynes·sec^−1^·cm^−5^. Fluid administration was highest from admission to the following morning, and by the fourth morning, the median fluid balance was negative (Fig. 1E). The mean noradrenaline dose was initially 0.08 μg·kg^−1^·min^−1^ and decreased to 0.02 μg·kg^−1^·min^−1^ during the study period (Fig. 1F).

### Circulatory state sequences and distribution

**Fig. 2:**
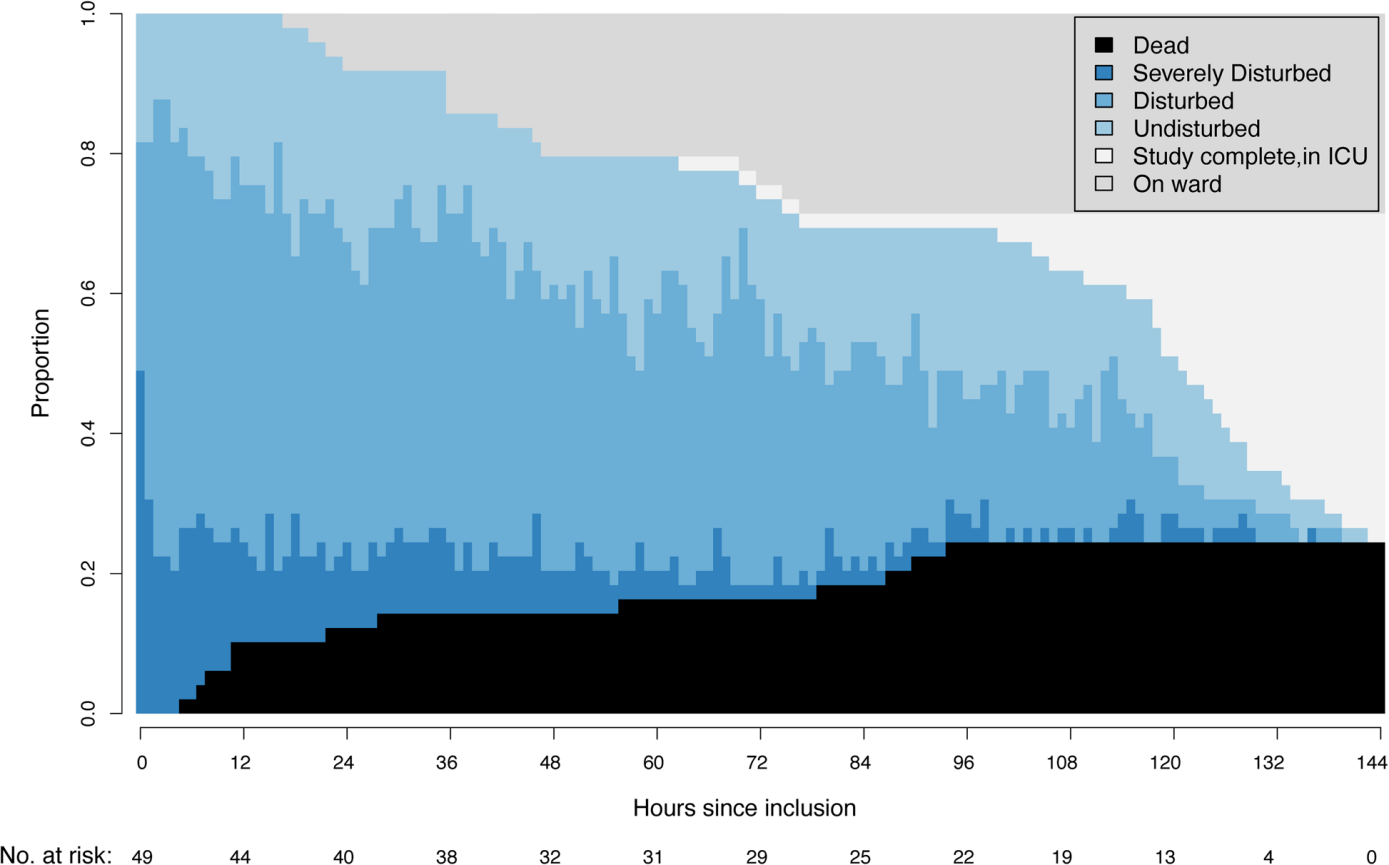
Distribution plot. Hourly distribution of circulatory states, including number at risk for state transition and coded absorbing states (i.e., death, discharge to the ward and still in the ICU but out of study). ICU: Intensive care unit

During the study period, 869 circulatory state transitions were recorded and analyzed. One patient was excluded from this analysis due to problems with data sampling. The hourly distributions of patients in each circulatory state, together with patients who died or were transferred out of the ICU, are shown in Fig. 2.

At hospital admission, approximately half of the patients were in a state of ‘severely disturbed’ circulation. Over time, circulation improved for most patients (Fig. 2). More patients entered the state of ‘disturbed’ circulation during the first 72 h than after. At the end of the study period, 14 (28%) patients had been transferred to the ward, 23 (46%) were still in ICU care, and 12 (24%) died (Fig. 2).

Hypotension, heart rate and dose of noradrenaline were the variables that most frequently “triggered” a change to a worse circulatory state (Supplementary Figure 2).

### Circulatory trajectories

**Fig. 3:**
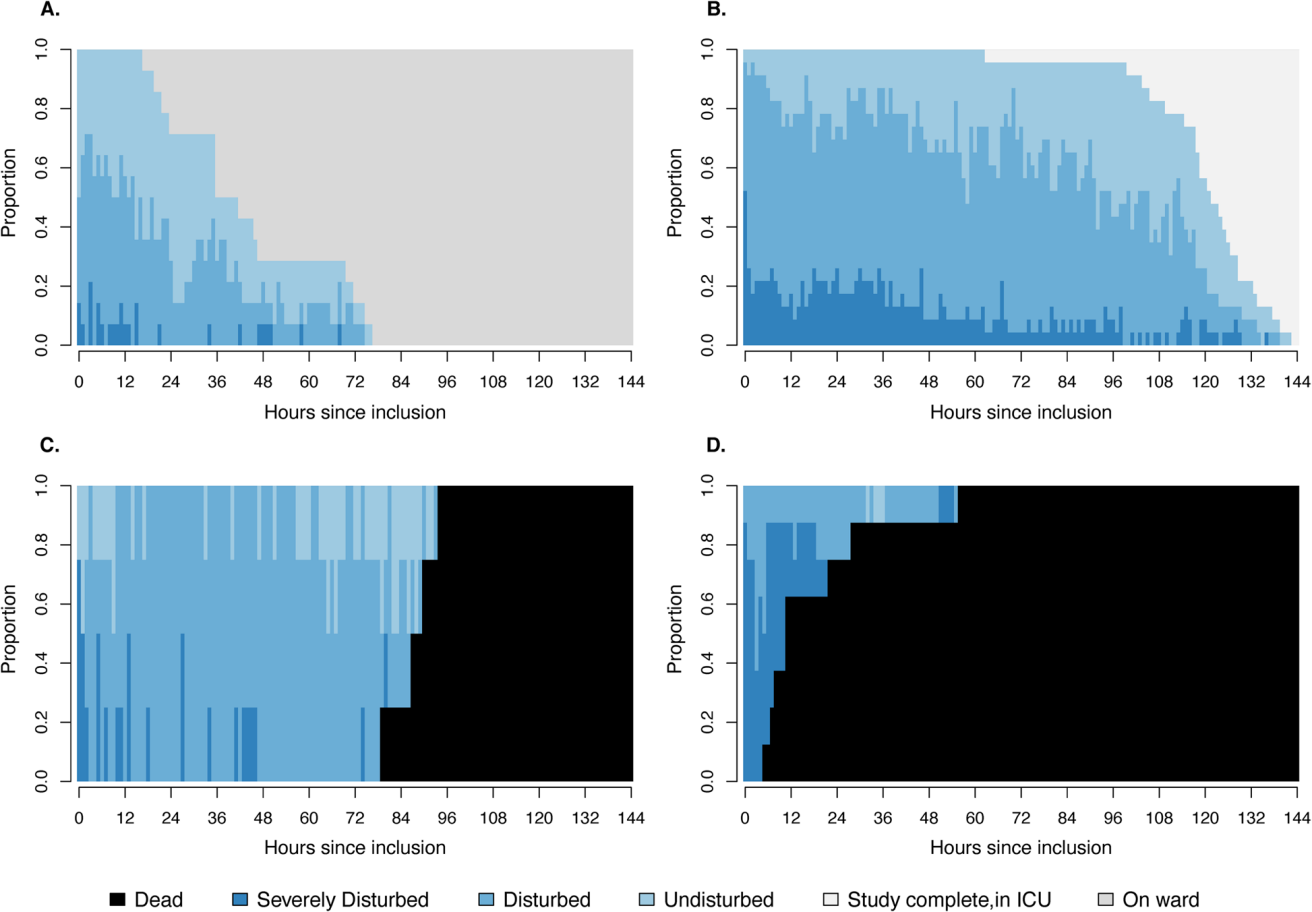
Distribution plot for clusters 1 to 4. Hourly distribution of circulatory states, including death, discharge to the ward and still in the ICU but out of study. **A** Cluster 1. **B** Cluster 2. **C** Cluster 3. **D** Cluster 4. ICU: Intensive care unit

We identified four typical clusters of circulatory trajectories after OHCA. ‘Cluster 1’ (28% of patients) describes a circulatory trajectory where most patients were stabilized within 48 h and transferred to a general ward (Fig. 3a). ‘Cluster 2’ was the dominant cluster (46% of patients) and showed a trajectory where the patients were mostly in the disturbed circulatory state and remained sedated and ventilated during the study period (Fig. 3b).

‘Cluster 3’ (8% of patients) describes a trajectory in predominantly disturbed circulatory states that ends in death within 96 h (Fig. 3c), whereas ‘Cluster 4’ (16% of patients) shows a more dramatic trajectory with patients in severe circulatory state until death, typically within 24 h (Fig. 3d).

In the multivariable analysis, base deficit at admission (OR 1.18 per mmol·L^−1^) was significantly associated with a less favorable cluster and thus a worse circulatory trajectory (Table 3). An initial shockable cardiac rhythm (ventricular fibrillation or tachycardia) was associated with a more favorable cluster (OR 0.07). Characteristics and sequence plots of the clusters are presented in Supplementary Table 1 and Supplementary Figure 3, respectively. The model did not violate the proportional odds assumption between the clusters (Χ^2^ = 0.15).

**Table 3:** Ordered logistic regression analysis of the association between cluster membership and demographic variables

Demographic variables	Univariable analysis	Multivariable analysis
	Odds ratio (95% CI)	Odds ratio (95% CI)
Age, per 5 years	1.06 (0.88—1.28)	1.17 (0.84—1.63)
Charlson Comorbidity Index, point	0.97 (0.76—1.23)	1.06 (0.70—1.61)
Initial shockable rhythm, yes	0.02 (0.004—0.14)	0.07 (0.01—0.46)
Time to ROSC, per 5 min	1.04 (1.01—1.09)	1.12 (0.88—1.42)
Base deficit at admission, per mmol/L	1.23 (1.10—1.37)	1.18 (1.03—1.35)
Circulatory shock^a^ in the ER, yes	5.64 (1.71—18.62)	1.93 (0.47—7.85)

In ordered logistic regression, the odds ratios among clusters are equal, and the odds ratio should be interpreted as the odds of a higher cluster than the compared cluster when the explanatory variable is increased by one unit and all other variables are held constant. Pseudo *R*^2^ = 0.30

*CI* Confidence interval, *ER* Emergency room, *ROSC* Return of spontaneous circulation

^a^Systolic blood pressure < 90 mmHg or in need of fluids and/or vasopressors to maintain systolic blood pressure > 90 mmHg

### Morbidity and mortality

During the first four days, the patients had median SOFA scores between 10 and 11 points, which improved on the fifth day to a median of 7.5 points. The most frequent organ system failures were circulatory, neurologic and respiratory (Supplementary Table 2).

Sixteen patients died within 180 days (Table 2). Six patients died within 48 h, predominantly from refractory circulatory shock or multiorgan failure (4 of 6 patients), while ten patients died later, mostly due to irreversible brain injury (8 of 10 patients). All eight patients who were awake at admission survived with good neurologic outcomes (defined as CPC 1). Twenty-two of the 26 patients who were comatose at admission and discharged alive from the hospital had good neurologic outcomes (CPC 1) after 180 days.

## Discussion

We found that after out-of-hospital cardiac arrest, patients had an overall two-phase circulatory development. Low CO and high SVR characterized the initial phase, whereas high CO and low SVR characterized the later phase. We identified four clusters of circulatory trajectories after OHCA. Multivariable analysis revealed that initial shockable rhythm was significantly associated with a favorable circulatory trajectory, while metabolic acidosis at admission was associated with an unfavorable circulatory trajectory.

Current AHA guidelines recommend MAP > 65 mmHg [5]. Patients included in our study had a median MAP between 70 and 75 mmHg during the study period. This was achieved by fluid and vasopressor administration. After liberal fluid resuscitation for the first 12 h, the need for fluids was gradually reduced in the following days. A similar pattern was evident for norepinephrine, where the mean dose was reduced after a few hours of intensive care. During the first 48 h, the CO increased concomitantly with a decrease in the calculated SVR. This pattern has previously been interpreted as resolving myocardial stunning, followed by peripheral vasodilatation due to systemic inflammation [2]. However, in this study, the median MAP and filling pressure were stable in the higher normal range, and the decrease in median SVR did not lead to an increase in vasopressor support or fluid resuscitation. Because CO and SVR are reciprocal values given constant arterial to venous pressure differences, the reduced calculated SVR might not be clinically relevant but rather an artifact due to increasing CO.

Seventy-two hours was found to be a “turning point” in circulatory stabilization. First, the majority of patients had reached a state of ‘undisturbed’ circulation by this time. Second, the majority of patients in this study achieved negative daily fluid balance between 72 and 96 h after cardiac arrest. In critically ill patients, persistent positive daily fluid balance beyond day four is associated with higher mortality [26, 27]. These observations suggest a stabilizing circulatory status within three days and are in accordance with findings by Laurent and coworkers [2].

We identified four clusters of circulatory trajectories. Three of the four clusters reached a finite state after 72 h: either stable and transferred to the ward (‘Cluster 1’) or dead (‘Cluster 3’ and ‘Cluster 4’). ‘Cluster 2’ remained in intensive care after 72 h. SOFA scores showed that most patients who remained in the ICU experienced multiorgan failure. However, 20 of 23 patients in ‘Cluster 2’ ultimately survived, and 18 of 23 patients had good neurologic outcomes (CPC 1). This observation supports that long-term intensive care treatment of OHCA patients is usually indicated, as the majority of patients, although critically ill, survived with a good cerebral outcomes.

Base deficit at admission was associated with an unfavorable circulatory trajectory, whereas initial shockable cardiac rhythm was associated with a favorable circulatory trajectory. Time to ROSC is a strong predictor for outcome [22], as seen in the univariable analysis. However, there is a high degree of collinearity between the initial shockable cardiac rhythm (i.e., ventricular tachycardia or fibrillation) and time to ROSC, and only the former was included in the final multivariable model [28, 29]. Bro-Jeppesen and coworkers have shown that increased lactate at admission is a strong predictor of vasopressor need [23]. This observation agrees with our findings, as both high lactate and metabolic acidosis at admission are indicative of “stressed metabolism” during the prehospital phase. Signs of stressed metabolism in combination with nonshockable rhythm, alternatively long time to ROSC, are suggestive of a high “ischemia–reperfusion burden” and thus a worse circulatory trajectory.

To make the result more clinically generalizable and to have a larger variability in independent predictors, thereby increasing the potential for identifying potential predictors, we included both awake and comatose patients. Awake patients usually experienced cardiac arrest, a short time to ROSC and excellent outcomes after hospital admission.

Age and comorbidities are usually associated with organ failure and mortality in an intensive care population [30] but were not significantly associated with a less favorable circulatory trajectory in our study. We observed a similar pattern of organ failure after OHCA as described by Roberts et al., with severe circulatory, respiratory and cerebral failures (SOFA score 3–4) and milder coagulation and kidney dysfunctions (SOFA score 1–2) [31]. Liver dysfunction was rare.

Sixteen of 42 (38%) patients who were comatose at hospital admission died within 180 days after OHCA. This is in line with previously reported mortality rates [32]. Furthermore, we found the same two-phase death pattern as previously described [33]; the early deaths were dominated by circulatory collapse and multiorgan failure, whereas later deaths were dominated by severe brain injury.

### Strengths and limitations

The strengths of this study are its prospective design, consecutive inclusion of patients, and data including central hemodynamic measurements being obtained continuously and frequently. We also recognize some potential limitations. First, this was a single-center study with a limited number of patients, which might limit the generalizability of the results and increase the probability of making a type-2 error. However, the number of circulatory state transitions was high (869 transitions) and was sufficient to perform analyses regarding circulatory trajectories. Second, the variables and thresholds used to define the circulatory categories have not been validated. However, no consensus exists on how to define circulatory instability; therefore, we utilized measurements that are routinely available in ICU patients and thresholds based on general guidelines. Finally, sequence analysis is a complex procedure. The penalty cost of sequence editing is debatable, and a different value could have resulted in a different pairwise matching and perhaps cluster membership. However, the four clusters described in this study seem clinically reasonable.

## Conclusions

Low CO and high SVR characterized the initial circulatory failure after OHCA. During the first 48 h, this pattern reversed to a high CO and low SVR. The majority of patients experienced circulatory stabilization within 72 h after cardiac arrest. We identified four clusters of patients with different severities of circulatory failure. Initial shockable cardiac rhythm was associated with a favorable circulatory trajectory, and low base excess at admission was associated with an unfavorable circulatory trajectory.

## Supplementary Information

**Additional file 1: Supplementary Figure 1.** Flowchart summarizing patient enrollment and exclusion. CA: Cardiac arrest. ICU: Intensive care unit. ECMO: Extracorporeal membranous oxygenation. OHCA: Out-of-hospital cardiac arrest. PAC: Pulmonary artery catheter. VAD: Ventricular assist device.

**Additional file 2: Supplementary Figure 2.** Heat-map showing which of the variables in the circulatory state model that categorizes the patient in a worse circulatory state. IABP: Intra-aortic balloon pump. MAP: Mean arterial pressure.

**Additional file 3: Supplementary Figure 3.** Sequence plot for cluster 1 to 4, showing sequences of longitudinal succession of circulatory states, i.e. trajectory, for every patient in the respective cluster. **A.** Cluster 1. **B.** Cluster 2. **C.** Cluster 3. **D.** Cluster 4. ICU: Intensive care unit.

**Additional file 4: Supplementary Table 1.** Demographic and mortality for cluster 1 to 4. * Systolic blood pressure <90 mmHg or in need of fluids and/or vasopressors to maintain systolic blood pressure >90 mmHg. † Comatose were patients that were intubated and gave no contact (GCS <8). ER: Emergency room. GCS: Glasgow coma scale. ROSC: Return of spontaneous circulation. SD: Standard deviation. SAPS: Simplified Acute Physiology Score.

**Additional file 5: Supplementary Table 2.** Sequential Organ Failure Assessment score. * In sedated patients daily Glasgow Coma Scale is based on pre-sedation score. SOFA: Sequential Organ Failure Assessment. Q1–Q3: first to third quartiles.

## Data Availability

The datasets used and/or analyzed during the current study are available from the corresponding author on reasonable request.

## Abbreviations

AHA: American Heart Association
CA: Cardiac arrest
CO: Cardiac output
CPC: Cerebral performance category
ECMO: Extracorporeal membranous oxygenation
ICU: Intensive care unit
ILCOR: International Liaison Committee on Resuscitation
MAP: Mean arterial pressure
MPAP: Mean pulmonary arterial pressure
OHCA: Out-of-hospital cardiac arrest
PAC: Pulmonary artery catheter
PCAS: Post-cardiac arrest syndrome
ROSC: Return of spontaneous circulation
SAPS-2: Simplified Acute Physiology Score 2
SD: Standard deviations
SOFA: Sequential Organ Failure Assessment
SVR: Systemic vascular resistance
VAD: Ventricular assist device
Q1–Q3: First to third quartiles
