# Supplementary figures and images for: Circulatory trajectories after out-of-hospital cardiac arrest: a prospective cohort study
Source: BMC Anesthesiol. 2021 Sep 8;21:219. doi: 10.1186/s12871-021-01434-2 (PMC8424149; doi:10.1186/s12871-021-01434-2)

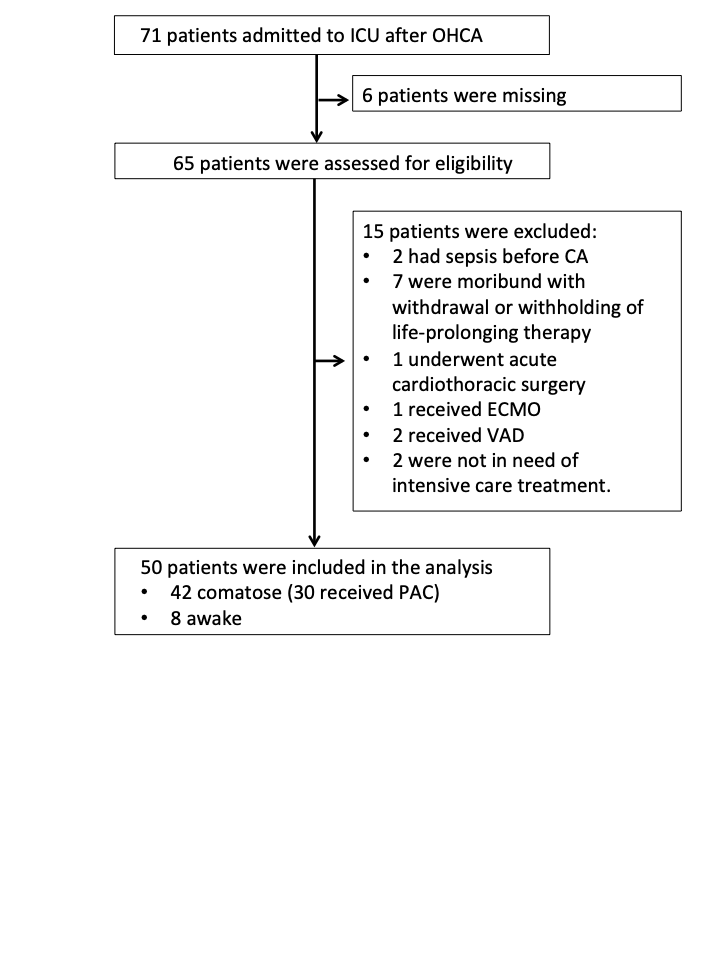

Supplement: Supplementary file 1 — Additional file 1: Supplementary Figure 1. Flowchart summarizing patient enrollment and exclusion. CA: Cardiac arrest. ICU: Intensive care unit. ECMO: Extracorporeal membranous oxygenation. OHCA: Out-of-hospital cardiac arrest. PAC: Pulmonary artery catheter. VAD: Ventricular assist device. [file 12871_2021_1434_MOESM1_ESM.tiff]

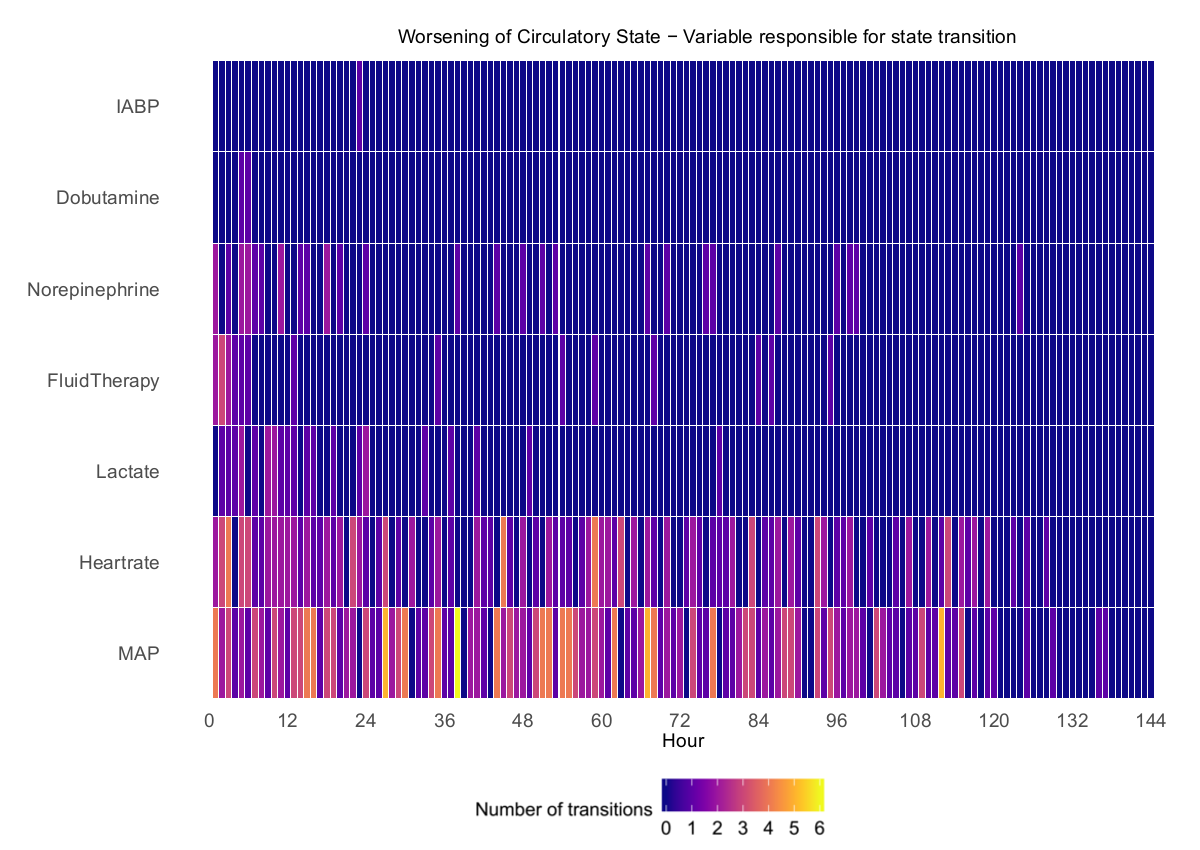

Supplement: Supplementary file 2 — Additional file 2: Supplementary Figure 2. Heat-map showing which of the variables in the circulatory state model that categorizes the patient in a worse circulatory state. IABP: Intra-aortic balloon pump. MAP: Mean arterial pressure. [file 12871_2021_1434_MOESM2_ESM.tif]

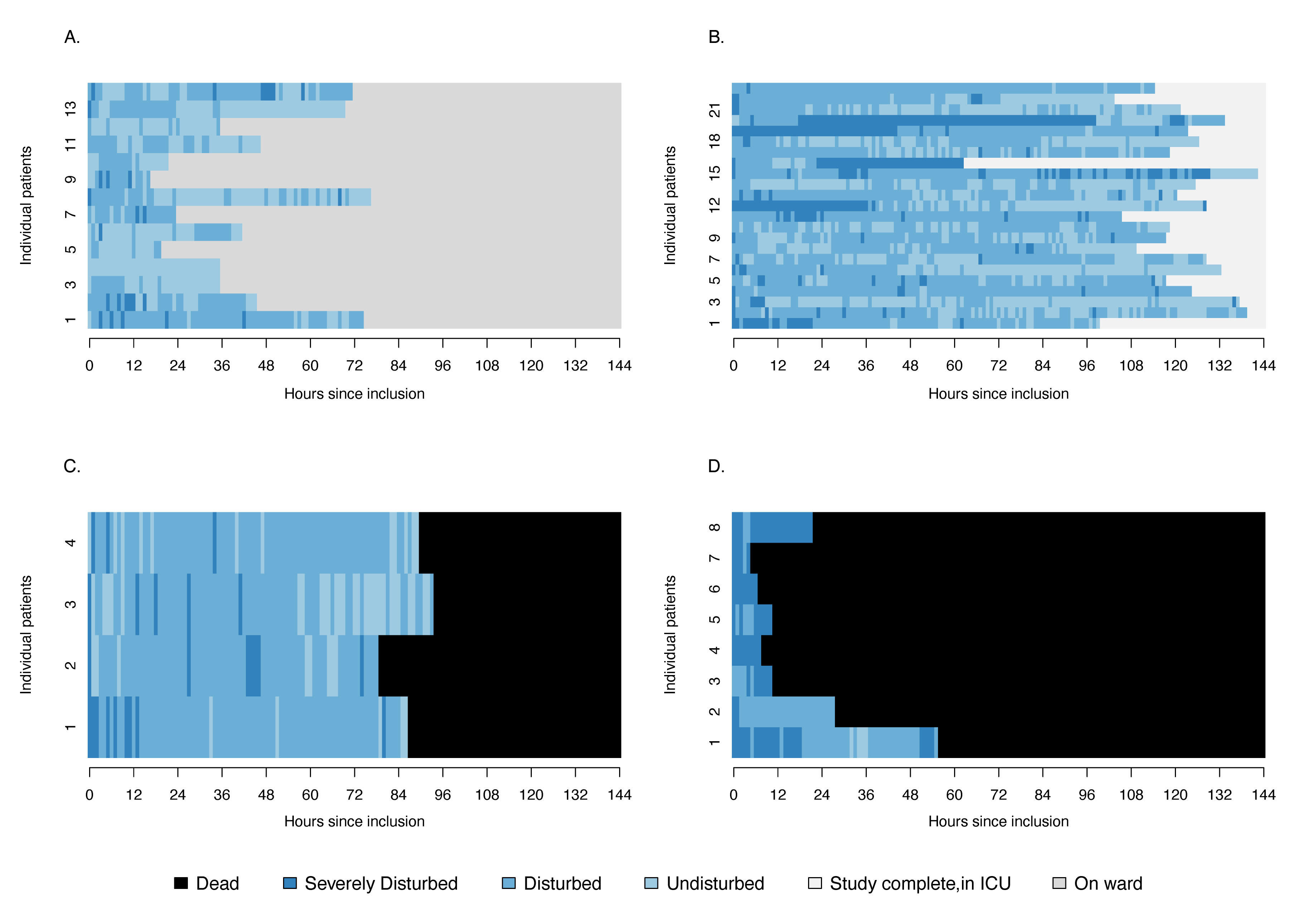

Supplement: Supplementary file 3 — Additional file 3: Supplementary Figure 3. Sequence plot for cluster 1 to 4, showing sequences of longitudinal succession of circulatory states, i.e. trajectory, for every patient in the respective cluster. A. Cluster 1. B. Cluster 2. C. Cluster 3. D. Cluster 4. ICU: Intensive care unit. [file 12871_2021_1434_MOESM3_ESM.tif]
