# Supplementary material for: Circulatory trajectories after out-of-hospital cardiac arrest: a prospective cohort study
Source: BMC Anesthesiol. 2021 Sep 8;21:219. doi: 10.1186/s12871-021-01434-2 (PMC8424149; doi:10.1186/s12871-021-01434-2)
Supplement: Supplementary file 4 — Additional file 4: Supplementary Table 1. Demographic and mortality for cluster 1 to 4. * Systolic blood pressure <90 mmHg or in need of fluids and/or vasopressors to maintain systolic blood pressure >90 mmHg. † Comatose were patients that were intubated and gave no contact (GCS <8). ER: Emergency room. GCS: Glasgow coma scale. ROSC: Return of spontaneous circulation. SD: Standard deviation. SAPS: Simplified Acute Physiology Score. [file 12871_2021_1434_MOESM4_ESM.docx]

| **Supplementary Table 1.** Demographic and outcome for cluster 1 to 4. | | | | |  | |
| --- | --- | --- | --- | --- | --- | --- |
|  |  |  |  |  |  | |
| **Characteristics of clusters** | **1** | **2** | **3** | **4** | **ANOVA*** | |
|  | n = 14 (28%) | n = 23 (46%) | n = 4 (8%) | n = 8 (16%) | *F* | *p* |
| Age, years, mean (sd) | 59 (16) | 65 (12) | 59 (13) | 64 (21) | 0.46 | 0.70 |
| Body mass index, mean (sd) | 26.4 (2.5) | 27.3 (4.8) | 34.3 (18.7) | 25.6 (4.2) | 1.73 | 0.17 |
| Charlson comorbidity index, mean (sd) | 3.5 (3.2) | 3.5 (1.7) | 2.7 (1.7) | 3.5 (2.6) | 0.13 | 0.94 |
|  |  |  |  |  |  |  |
| Shockable initial rhythm, no. (%) | 13 (92) | 22 (95) | 2 (50) | 1 (12) | 19.0 | 0.00^†^ |
| Time to ROSC, min., mean (sd) | 17.3 (11.4) | 27.8 (15.8) | 31.5 (6.13) | 31.8 (19) | 19.0 | 0.00^†^ |
| Presumed cardiac etiology, no. (%) | 13 (92) | 22 (95) | 3 (75) | 3 (37.5) | 7.2 | 0.00^‡^ |
| Circulatory shock in the ER^§^, no. (%) | 1 (8) | 10 (43) | 1 (25) | 6 (75) | 4.3 | 0.01^¶^ |
| Comatose at admission^**^, no. (%) | 7 (50) | 23 (100) | 4 (100) | 8 (100) | 10.7 | 0.00^††^ |
|  |  |  |  |  |  |  |
| Initial pH, mean (sd) | 7.3 (0.07) | 7.18 (0.14) | 7.08 (0.07) | 7.06 (0.16) | 6.3 | 0.00^‡‡^ |
| Initial base excess, mmol/L, mean (sd) | -6.8 (4.8) | -9.3 (5.2) | -16 (2.8) | -16.3 (5.9) | 7.6 | 0.00^§§^ |
| Initial lactate level, mmol/L, mean (sd) | 4.8 (3.3) | 5.4 (3.4) | 10.9 (1.1) | 11.6 (4.1) | 9.9 | 0.00^†^ |
|  |  |  |  |  |  |  |
| SAPS II, mean (sd) | 42.5 (17.3) | 68.3 (11.2) | 60 (3.1) | 78.6 (13.3) | 9.9 | 0.00^†^ |
|  |  |  |  |  |  |  |
| 30 days mortality, no. (%) | 0 (0) | 3 (13) | 4 (100) | 8 (100) | 44.8 | 0.00^†^ |
| 180 days mortality, no. (%) | 1 (7) | 3 (13) | 4 (100) | 8 (100) | 30.1 | 0.00^†^ |
|  |  |  |  |  |  | |

* If one-way ANOVA indicated a significant difference between groups, Tukey’s method were used to determine which of the groups that were significantly different.

† Cluster 3 and 4 is significantly different from cluster 1 and 2.

‡ Cluster 4 is significantly different from cluster 1 and 2.

§ Systolic blood pressure <90 mmHg or in need of fluids and/or vasopressors to maintain systolic blood pressure >90 mmHg.

¶ Cluster 4 and 1 are significantly different.

** Glasgow coma scale <8 and intubated.

†† Cluster 1 is significantly different from all other clusters.

‡‡ Cluster 1 is significantly different from cluster 3 and 4.

§§ Cluster 4 is significantly different from cluster 1 and 2, and cluster 3 is significantly different from cluster 1.

ER: Emergency room. ROSC: Return of spontaneous circulation. SD: Standard deviation. SAPS: Simplified Acute Physiology Score
