# Supplementary material for: Circulatory trajectories after out-of-hospital cardiac arrest: a prospective cohort study
Source: BMC Anesthesiol. 2021 Sep 8;21:219. doi: 10.1186/s12871-021-01434-2 (PMC8424149; doi:10.1186/s12871-021-01434-2)
Supplement: Supplementary file 5 — Additional file 5: Supplementary Table 2. Sequential Organ Failure Assessment score. * In sedated patients daily Glasgow Coma Scale is based on pre-sedation score. SOFA: Sequential Organ Failure Assessment. Q1–Q3: first to third quartiles. [file 12871_2021_1434_MOESM5_ESM.docx]

|  |  | |  |  |  |  |  |  |
| --- | --- | --- | --- | --- | --- | --- | --- | --- |
| **Supplementary Table 2.** Sequential Organ Failure Assessment | | | | | | | | |
| **Organ failure** | | | | **Day 1** | **Day 2** | **Day 3** | **Day 4** | **Day 5** |
|  |  | |  | n = 50 | n = 44 | n = 35 | n = 29 | n = 24 |
| **Total score**, median (Q1–Q3) | | | | 11 (9-12) | 10 (7-12) | 11 (7-12) | 10 (7-12) | 10 (6-11) |
|  | **Respiratory score,** median (Q1–Q3) | | | 3 (2-4) | 2 (2-3) | 3 (2-3) | 2 (2-3) | 2,5 (2-3) |
|  |  | Dysfunction (SOFA score 1 - 2), no. (%) | | 15 (30) | 20 (45) | 14 (40) | 15 (52) | 12 (50) |
|  |  | Failure (SOFA score 3 - 4), no. (%) | | 31 (62) | 21 (49) | 20 (57) | 13 (44) | 12 (50) |
|  | **Circulatory score,** median (Q1–Q3) | | | 4 (3-4) | 4 (3-4) | 3 (0-4) | 3 (1-3) | 3 (0,5-3) |
|  |  | Dysfunction (SOFA score 1 - 2), no. (%) | | 3 (6) | 1 (2) | 2 (6) | 3 (10) | 5 (21) |
|  |  | Failure (SOFA score 3 - 4), no. (%) | | 42 (84) | 37 (84) | 24 (68) | 19 (65) | 13 (54) |
|  | **Hepatic score,** median (Q1–Q3) | | | 0 (0-0) | 0 (0-0) | 0 (0-0) | 0 (0-0) | 0 (0-0) |
|  |  | Dysfunction (SOFA score 1 - 2), no. (%) | | 5 (10) | 5 (11) | 7 (20) | 7 (24) | 4 (16) |
|  |  | Failure (SOFA score 3 - 4), no. (%) | | 0 (0) | 0 (0) | 0 (0) | 0 (0) | 0 (0) |
|  | **Renal score,** median (Q1–Q3) | | | 0 (0-1) | 0 (0-1) | 0 (0-1) | 0 (0-0) | 0 (0-0) |
|  |  | Dysfunction (SOFA score 1 - 2), no. (%) | | 17 (34) | 15 (34) | 11 (32) | 5 (17) | 3 (12) |
|  |  | Failure (SOFA score 3 - 4), no. (%) | | 0 (0) | 0 (0) | 1 (3) | 2 (7) | 2 (8) |
|  | **Coagulation score,** median (Q1–Q3) | | | 0 (0-0) | 0 (0-0.5) | 1 (0-1) | 1 (0-1) | 1 (0-1) |
|  |  | Dysfunction (SOFA score 1 - 2), no. (%) | | 3 (6) | 11 (25) | 20 (57) | 16 (55) | 16 (66) |
|  |  | Failure (SOFA score 3 - 4), no. (%) | | 0 (0) | 0 (0) | 0 (0) | 0 (0) | 0 (0) |
|  | **Cerebral** **score***, median (Q1–Q3) | | | 4 (4-4) | 4 (1-4) | 4 (3-4) | 4 (3-4) | 4 (2-4) |
|  |  | Dysfunction (SOFA score 1 - 2), no. (%) | | 5 (10) | 5 (11) | 4 (11) | 6 (21) | 6 (25) |
|  |  | Failure (SOFA score 3 - 4), no. (%) | | 43 (86) | 32 (72) | 27 (78) | 22 (75) | 16 (66) |

* In sedated patients daily Glasgow Coma Scale is based on pre-sedation score.

SOFA: Sequential Organ Failure Assessment. Q1–Q3: first to third quartiles
